# Efficacy and safety of electrical moxibustion for knee osteoarthritis: study protocol for a randomized controlled trial

**DOI:** 10.1186/s13063-018-2514-x

**Published:** 2018-03-05

**Authors:** Ha-Ra Kang, Chan-Yung Jung, Seung-Deok Lee, Kyung-Ho Kim, Kap-Sung Kim, Eun-Jung Kim

**Affiliations:** 1 Department of Acupuncture & Moxibustion, Dongguk University Ilsan Oriental Hospital, Dongguk-ro, Ilsandong-gu, Goyang-si, Gyeonggi-do Republic of Korea; 2 College of Korean Medicine, Dongguk University, Dongdae-ro, Gyeongju-si, Gyeongsangbuk-do Republic of Korea; 3 Department of Acupuncture & Moxibustion, Dongguk University Bundang Oriental Hospital, Bundang-gu, Seongnam-si, Gyeonggi-do Republic of Korea

**Keywords:** Knee osteoarthritis, Electrical moxibustion, Moxibustion, Randomized controlled trial

## Abstract

**Background:**

Knee osteoarthritis (KOA) is a significant health issue because it causes pain and functional limitation. Many studies have reported that moxibustion, a treatment in traditional Korean medicine, is effective in treating KOA. However, conventional moxibustion produces smoke, harmful gases, and odors that can adversely affect the eyes, skin, and throat. It is also difficult to control the intensity of stimulation in conventional moxibustion. An electrical moxibustion device was developed to circumvent these problems, but there are few studies of that device. We will evaluate the efficacy and safety of electrical moxibustion as a treatment for KOA, and compare it with traditional indirect moxibustion and usual care.

**Methods:**

This is a multicenter, randomized, open, assessor-blinded, parallel-group clinical trial. A total of 138 eligible participants with KOA will be randomly allocated into three groups (electrical moxibustion, traditional indirect moxibustion, or usual care) with a 1:1:1 ratio. Participants in each moxibustion group will receive 12 sessions of moxibustion treatment at 6 acupoints (ST36, ST35, ST34, SP9, EX-LE4, SP10) plus up to 2 points of “ashi”, if needed, over a period of 6 weeks (2 sessions per week). A specifically designed device that provides thermal stimulation using electrical energy will be used for the electrical moxibustion group. Participants in the usual care group will receive usual treatment and self-care. The primary outcome measure is change in pain on a numerical rating scale (NRS) from week 1 to week 6. The secondary outcome measures are pain assessed on a visual analog scale (VAS), the Korean version of the Western Ontario and McMaster osteoarthritis index (K-WOMAC), patient global assessment (PGA), and the European quality of life five dimension five level scale (EQ-5D-5 L). Safety will be assessed by monitoring adverse events at each visit. Follow-up measurements will be performed at 12 weeks after baseline measurements.

**Discussion:**

This trial will provide evidence on the efficacy and safety of electrical moxibustion as a treatment for KOA.

**Trial registration:**

ClinicalTrials.gov, NCT03287570. Registered on 19 September 2017.

**Electronic supplementary material:**

The online version of this article (10.1186/s13063-018-2514-x) contains supplementary material, which is available to authorized users.

## Background

Osteoarthritis is a degenerative joint disease caused by loss of articular cartilage [1]. As part of the weight-bearing peripheral and axial joints, the knees are commonly affected by osteoarthritis [2]. The worldwide prevalence of osteoarthritis continues to increase as societies age [3], so it has an increasing impact on society because it reduces the ability to work and increases medical costs [4].

Oral medications, such as non-steroidal anti-inflammatory drugs (NSAIDs) and acetaminophen, intra-articular injections, and physical therapy are generally used to treat knee osteoarthritis (KOA) [5]. But these treatments only provide modest short-term relief from pain, and do not inhibit disease progression. Furthermore, these treatments may lead to adverse gastrointestinal, cardiovascular, and renal events [6]. Therefore, many patients with KOA are seeking complementary and alternative treatments.

Traditional Korean medicines, such as acupuncture [7, 8], moxibustion [9, 10], and herbal medications [11], are widely used to manage KOA, and many studies have reported positive outcomes. There is great interest in moxibustion, the thermal and chemical stimulation from burning herbs at acupoints [12].

However, it is difficult to control the intensity of stimulation in conventional moxibustion due to variations in the types and amounts of herbs. If thermal stimulation is too strong, it causes pain and leaves scars from burns [13]. In addition, the process of combusting herbs produces smoke and this may lead to discomfort in patients and practitioners, such as dry eyes, dry skin, dry throat and coughing, and even reactive airway dysfunction syndrome in severe cases [14–16]. Odors from moxibustion can also cause headache, dizziness, and nausea [17].

An electrical moxibustion (EM) device circumvents many of the problems of conventional moxibustion, but there are few studies of such devices. We will conduct a clinical trial to investigate the efficacy and safety of EM as a treatment for KOA compared with traditional indirect moxibustion (TIM) and usual care.

## Methods/design

### Objective

The aim of this trial is to assess the efficacy and safety of EM compared with TIM and usual care in patients with KOA.

### Design and setting

This is a multicenter, randomized, open, assessor-blinded, parallel-group clinical trial. Eligible participants who meet the clinical criteria for KOA of the American College of Rheumatology (ACR) [18] will be enrolled. Two clinical research centers in Korea will participate: Dongguk University Bundang Oriental Hospital and Dongguk University Ilsan Oriental Hospital. A total of 138 participants with KOA will be randomly assigned to one of three groups (EM, TIM, or usual care) with a 1:1:1 ratio.

**Fig. 1:**
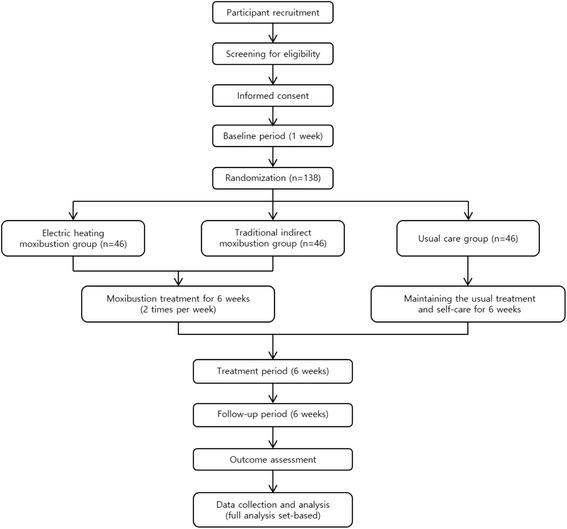
Procedures used for recruitment, randomization, treatment, and assessment. Participants with diagnoses of KOA will be recruited from two centers. The 2 treatment groups will receive 2 sessions per week of moxibustion for 6 weeks, followed by a 6 week follow-up period. Outcome measures will be determined at every visit

**Fig. 2:**
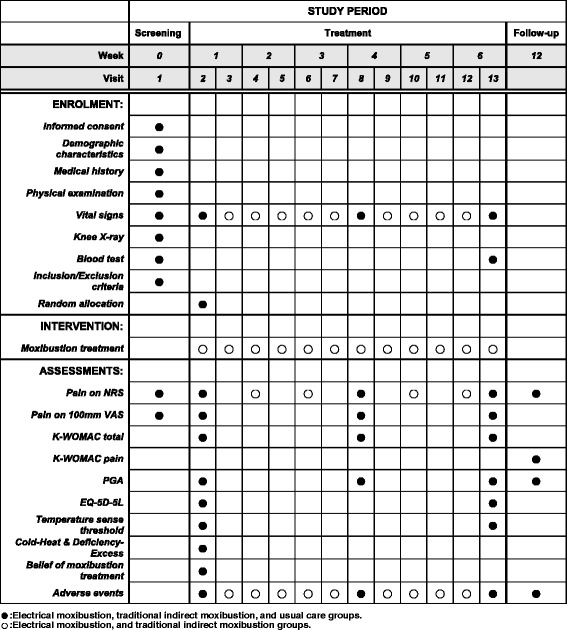
Schedule to be used for enrolment, intervention, and assessments. NRS, Numerical rating scale; VAS, Visual analogue scale; K-WOMAC, Korean version of Western Ontario and McMaster Universities osteoarthritis index; PGA, Patients global assessment; EQ-5D-5L, European quality of life five dimension five level scale

The study period is 12 weeks, and consists of a 6-week treatment phase and a 6-week follow-up phase. After randomization, participants in the EM and TIM groups will receive 12 sessions of treatment over 6 weeks. Participants in the usual care group will be assessed at 1, 4, and 6 weeks after baseline. Follow-up assessment by telephone will be conducted at 6 weeks after the end of treatment for all groups. Figure 1 outlines the trial procedures and Fig. 2 shows the schedules for enrollment, intervention, and assessments. The SPIRIT checklist is provided in Additional file 1.

### Study subjects

#### Inclusion criteria

All enrolled participants will be 40–70 years old, have KOA according to ACR criteria [18] (pain within the previous 6 months in one or both knees during weight loading, which has an average intensity of 40 mm or more on a 0–100 mm visual analog scale (VAS)), ability to distinguish temperature changes, willingness to participate in this clinical trial and to sign the informed consent agreement, and being reliable and willing to follow the restrictions for 3 months.

#### Exclusion criteria

Subjects will be excluded if they have any of the following conditions: traumatic injury that might be related to the current knee pain within the previous 6 months, knee surgery within the previous 6 months, use of intra-articular injections in the knee joint(s) within the previous 3 months, physical examination or radiographic results indicating the presence of rheumatoid arthritis, an autoimmune disease, or another type of inflammatory arthritis of the knee(s), a physical or psychiatric disorder that may affect moxibustion treatment, or a neurological disorder (including paralysis symptoms) that could affect local or general sensation. We also excluded pregnant or lactating women, those afraid of moxibustion treatment or who expected adverse effects, and those who the researcher determined were inappropriate for participation.

### Sample size

Sample size was estimated as described previously [19], which considered a change in the population mean on the numerical rating scale (NRS) for each group (1, EM group; 2, TIM group; 3, usual care group). The null and alternative hypotheses are:

$$ {H}_{01}:{\mu}_1-{\mu}_2=0,\kern0.5em {H}_{02}:{\mu}_1-{\mu}_3=0 $$

$$ {H}_{11}:{\mu}_1-{\mu}_2\ne 0,\kern0.5em {H}_{12}:{\mu}_1-{\mu}_3\ne 0 $$

H01 : the first null hypothesis

H02 : the second null hypothesis

H11 : the first alternative hypothesis

H12 : the second alternative hypothesis

μ1, μ2, μ3 : the true mean change of pain on the NRS before and after treatment (1: EM, 2: TIM, 3 : usual care)

We assumed that the true mean differences in pain on the NRS before and after treatment with EM and TIM were 12.25 (standard deviation (SD) 18.52) and 1.4 (SD 16.27), respectively. Thus, we calculated the sample size needed to achieve 80% power at a significance level of 0.05 using the following equation:

$$ n=\frac{\left({\sigma}_1^2+{\sigma}_2^2\right){\left({Z}_5+{Z}_{\upbeta}\right)}^2}{{\left({\mu}_1-{\mu}_2\right)}^2}\approx 41 $$

σ1, σ2 : the true standard deviation (1 : EM, 2 : TIM)

α : significance level

β : power

Z : points on normal distribution to give required power and significance

The mean difference between the usual care group and the EM and TIM groups is expected to be larger than that between the EM and TIM groups. Thus, sample size was estimated larger because of using the mean difference between the EM and TIM groups.

Thus, the estimated sample size was 41 participants per group. Considering an anticipated dropout rate of 10%, we plan to include a total of 138 participants, with 69 participants per institution.

### Recruitment

Participants will be recruited from outpatients attending the two hospitals. Printed recruitment posters will be distributed in each hospital and placed on bulletin boards and websites. The posters will provide a brief description of the trial, the details of treatments offered to eligible participants, and contact information. We will also advertise in local newspapers. Participants who are interested in participating will be able to contact the researcher directly.

Potential participants will be screened, receive explanations of the trial, and then sign informed consent agreements. Those who meet the selection criteria will receive baseline assessments, and demographic and general medical data will be collected, including medication use, vital signs, physical examination data, radiographic data, and laboratory data.

### Randomization and blinding

Eligible participants will be randomly assigned to one of three groups (EM, TIM, or usual care) with a 1:1:1 ratio. Randomization will be performed by an independent statistician with no clinical involvement in this trial, by use of a computerized random number generator through the stratified block method of SAS (SAS Institute Inc., Cary, NC, USA). The random numbers will be concealed using sequentially numbered, opaque, sealed envelopes. After the enrolled participants have completed all baseline assessments, the envelopes will be provided to doctors of traditional Korean medicine who will perform the moxibustion treatments. Allocation concealment will not be revealed until the final data analysis report is completed. This is an open clinical trial. Thus, the researcher who is blinded to the allocations will not participate in the treatment, but will perform the outcome assessment to reduce selection bias.

### Interventions

**Fig. 3:**
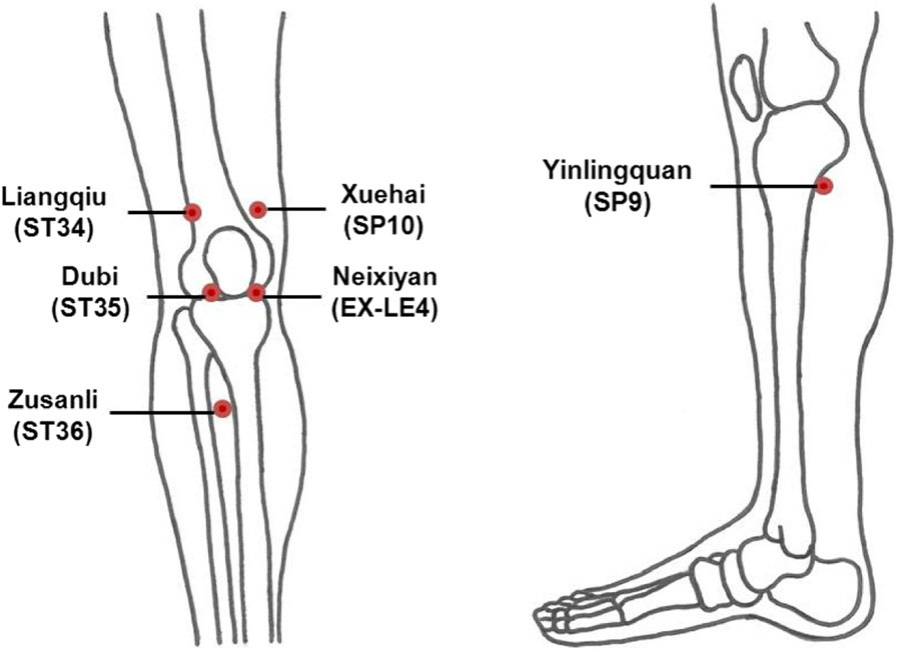
Acupoints used in the trial. The lines refer to acupoints of ‘Zusanli’ (ST36), ‘Dubi’ (ST35) and ‘Liangqiu’ (ST34) on the lateral side of the knee, and ‘Yinlingquan’ (SP9), ‘Neixiyan’ (EX-LE4), and ‘Xuehai’ (SP10) on the medial side of the knee

The treatment will be applied in 12 sessions, twice per week for 6 weeks. In the EM and TIM groups, moxibustion on the affected knee(s) will be provided at six standard acupuncture points: Zusanli (ST36), Dubi (ST35), Liangqiu (ST34), Yinlingquan (SP9), Neixiyan (EX-LE4), Xuehai (SP10) (Fig. 3), plus up to 2 points of “ashi”’, if needed, according to the determination of the practitioners. Treatments will be provided bilaterally in participants with pain in both knees. The treatment acupoints were selected based on a consensus of doctors of traditional Korean medicine, a text book, literature reviews, and additional studies [10, 19, 20]. The participants will be asked to wear loose clothing for easy exposure of the knee joints, and to maintain a comfortable supine position during treatment.

Participants in all groups will receive an educational leaflet that provides basic information about KOA, including its definition, pathology, current treatment options, instructions for daily activities, and advice to prevent exacerbation of symptoms. In addition, participants will be instructed in exercises to stretch the hamstrings and calf muscles and to strengthen muscles related to function of knees.

#### Electrical moxibustion

**Fig. 4:**
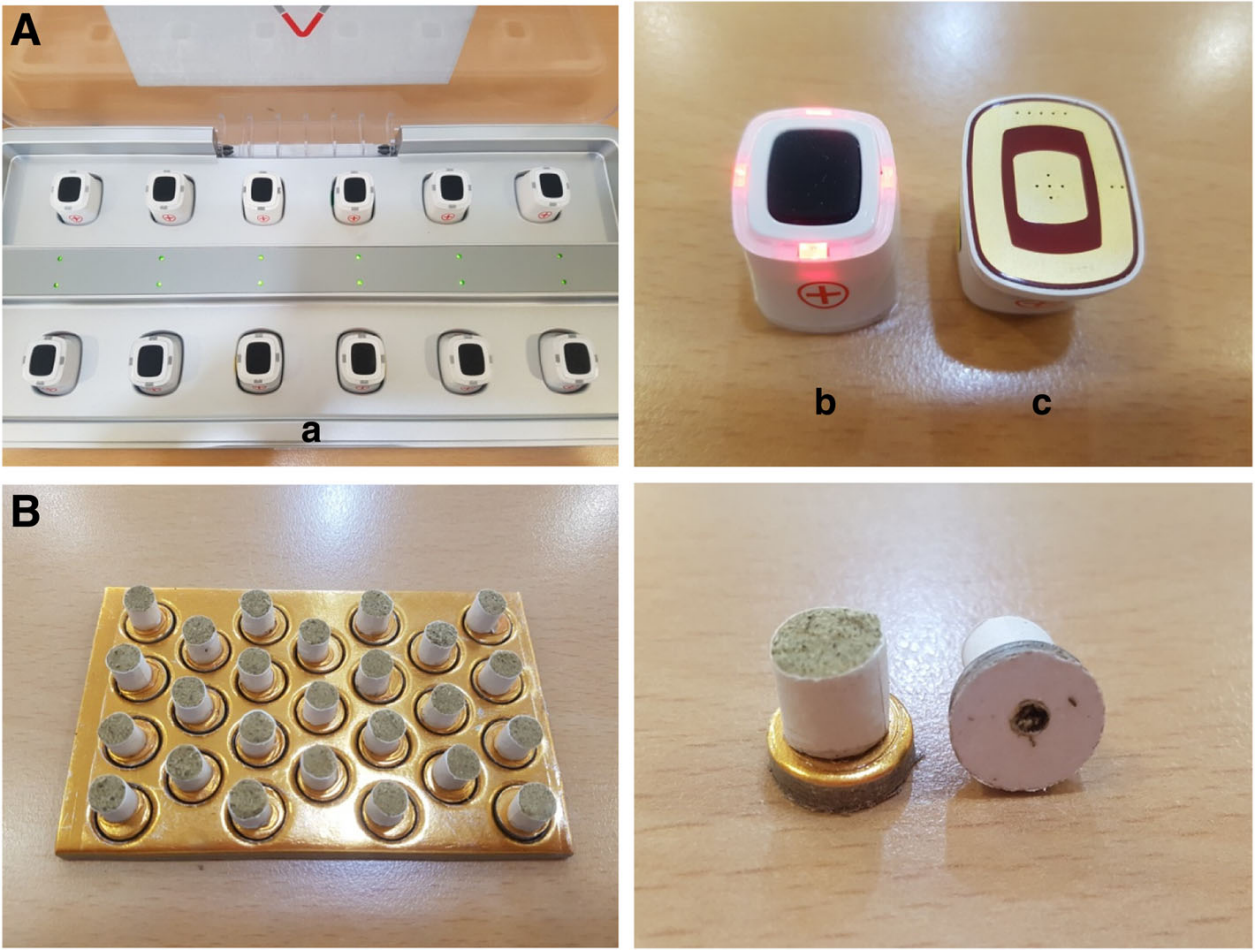
Moxibustion devices. **A** Electrical moxibustion device, showing the twelve heating units in the charging equipment (**a**), the power unit (**b**), and the bottom of the unit with the electrically heated board (**c**). **B** Traditional indirect moxibustion device

A specifically designed device (Cettum, K-medical Co., Korea) (Fig. 4) will be used to treat participants in the EM group. This device provides thermal stimulation using electricity and consists of heating units and charging equipment. After the heating units are attached to the skin using medical tape, the practitioner will press a button on top of the heating unit. As the color of the light emitting diode (LED) on the margins of the button changes, the temperature will adjust itself. When the temperature reaches 45 ± 1 °C, similar to the peak temperature of TIM, it will remain stable and then gradually decrease. If a participant complains of unbearable pain or heat, the attached point can be moved by up to 1 cm, according to the discretion of the practitioner. The expected total time of each treatment session will be 15 min.

#### Traditional indirect moxibustion group

Participants in the TIM group will be treated using mugwort, with a paper cylinder in which an adhesive on the bottom attaches to the skin (Taekeukttum, Haenglim Seowon Medical Co., China) (Fig. 4). The top of the moxibustion is combusted for approximately 5 to 10 s, and then attached to each point. When a participant reports that the heat tolerance has reached its maximum, the practitioner will place a new moxibustion at the same point. A total of three moxibustion cones will be applied to each point per treatment session. The expected total time of each treatment session will be 15 min.

#### Usual care group

Participants in this group will maintain the usual treatment and self-care.

#### Combined medication

Medications that were taken prior to entering the trial will be continued if they were taken for more than 1 month or if the researchers considered them unlikely to affect the results. Short-term use of new medications for treating new disorders or adverse events from treatment will be administered following consultation with the researchers. Detailed information on medication use will be documented in the case report forms (CRFs). Surgical interventions, intra-articular injection, newly administered antidepressant, anticonvulsant, cyclobenzaprine, psychotropic drugs, narcotic analgesics, or traditional Korean medical treatments (such as acupuncture and herbal medicines) to relieve knee pain will not be permitted.

### Quality assurance

Moxibustion will be performed by doctors of traditional Korean medicine who are certified by the Korean Ministry of Health and Welfare, are graduates of a 6-year full-time course in traditional Korean medicine taught as a college program, and have more than 1 year of postgraduate clinical experience. All practitioners, assessors, and assistants will receive training through a workshop, before the beginning of the trial. The workshop will provide training on the diagnosis of KOA, inclusion and exclusion criteria, standard operation procedures, location of acupoints, manipulation techniques of moxibustion devices, and outcome measures. The practitioners will be thoroughly checked prior to the start of the trial, and the treatment will be performed under the supervision of professors who specialize in acupuncture and moxibustion.

### Outcome assessments

#### Primary outcome

The primary outcome measure will be the mean change in pain on the NRS from week 1 (before treatment) to week 6. Participants in the EM and TIM groups will be asked to indicate a number between 0 and 100 that best describes the intensity of pain every week and at week 12 (follow-up check): 0 usually represents “no pain at all”, whereas 100 represents “the worst pain imaginable” [21]. Participants in the usual care group will be assessed using the same method at weeks 1, 4, 6, and 12.

#### Secondary outcomes

Secondary outcomes are:

- A 100-mm pain visual analogue scale (VAS) score: the pain VAS consists of a straight line with the endpoints defining extreme limits, in which 0 represents no pain and 100 the most extreme pain [22]. The participant will mark a point on this line that best indicates the pain level at weeks 1, 4, and 6.
- Western Ontario and McMaster Universities osteoarthritis index (WOMAC): this scale, which is widely used to evaluate the condition of patients with KOA, has 24 questions that assess disability. It has three subscales that measure pain (5 questions), stiffness (2 questions), and physical function (17 questions) about KOA. Each answer has a scale ranging from 0 (no symptoms) to 3 (maximum symptoms) [23]. There is a Korean version of the WOMAC (K-WOMAC), and Bae et al. confirmed its reliability, validity, and responsiveness [24]. We will use this scale to evaluate participants at weeks 1, 4, and 6.
- Patient global assessment (PGA): the PGA score is a self-reported 5-point measurement used to evaluate overall improvement after treatment. Participants will evaluate their improvement from baseline by selecting one of five options: much improved, minimally improved, no change, minimally worse, or much worse [25] at weeks 1, 4, 6, and 12.
- European quality of life five dimension five level scale (EQ-5D-5 L): this includes two main components: a descriptive scale and a VAS. The descriptive scale defines health-related quality of life (HRQoL) in 5 dimensions (mobility, self-care, usual activities, pain/discomfort, and anxiety/depression), in which each dimension has 5 levels of severity (no problems, slight problems, moderate problems, severe problems, and extreme problems). Participants are asked to indicate on the VAS how they feel on a scale of 0–100, where 0 indicates severe health problems and 100 represents excellent health [26]. Participants will be assessed using this scale at weeks 1 and 6.

### Dropouts

Participants who meet any of the following criteria will be excluded from the trial, and the specific reasons will be fully recorded: withdrawal of consent; new surgical intervention, injection, or oral drug for treatment of KOA during the clinical trial period; receiving additional traditional Korean medical treatments, such as acupuncture, moxibustion, and herbal medicine for KOA during the clinical trial period; receiving fewer than 10 moxibustion treatments or not participating in the follow-up; experiencing severe adverse events making further inclusion in the trial unsustainable; and if the researcher determines that further participation is inappropriate.

### Data management and monitoring

Upon conclusion of the treatment period, data will be completed and recorded on the original CRFs. No record will be missed or omitted, and the primary input of the data will not be permitted to be changed. Any corrections should be explained in the appended notes signed and dated by the researcher. Also, all data will be entered in an electronic database, following a standard data management protocol for data entry.

To protect confidentiality, written data will be stored in locked filling cabinets at the study sites with access limited to the researchers. Electronic data will be stored in a password-protected computer. After the trial, all documents will be preserved in the secure research archives at Dongguk University Oriental Hospital, and will only be available to the research team. All participants will have access to their personal data and the final study results.

Regular monitoring that will be clarified in a standard operating procedure will be conducted by an independent data monitoring committee. Monitors will evaluate whether the CRFs are properly written and whether the recruitment and intervention procedures are adequately performed according to the protocol.

### Statistical analysis

An independent statistician blinded to group allocation will perform statistical analysis on the full analysis set (FAS) and will also perform a pre-protocol (PP) analysis. Missing data will be replaced according to the principle of the last observation carried forward (LOCF) method.

Demographic and other baseline values will be analyzed using descriptive statistics for each group. For comparison of characteristics among the three groups, continuous data will be expressed as mean and SD and analyzed using analysis of variance (ANOVA) or the Kruskal-Wallis test. Categorical data will be presented as frequency and percentage and analyzed using chi-squared test or Fisher’s exact test.

The primary endpoint is the mean change in pain on the NRS from week 1 to week 6. To test the significance of changes in pain on the NRS between groups, we formulated two sets of hypotheses.

The first null hypothesis (H_01_) is that the EM and TIM groups have no difference in the true mean change of pain on the NRS before and after treatment. The first alternative hypothesis (H_11_) is that the EM and TIM groups have a difference in the true mean change in pain identified on the NRS before and after treatment.

The second null hypothesis (H_02_) is that the EM and usual care groups have no difference in the true mean change in pain on the NRS before and after treatment. The second alternative hypothesis (H_12_) is that the EM and usual care groups have a difference in the true mean change in pain on the NRS before and after treatment.

ANOVA or the Kruskal-Wallis test will be used to determine the significance of differences in the measured values before and after treatment in each group. To compare the three groups, analysis of covariance (ANCOVA) will be performed for the primary endpoint, with a fixed effect for the treatment group. Baseline pain on the NRS, age, Kellgren-Lawrence grade, and duration of KOA symptoms will be considered as covariates in this analysis. A *p* value below 0.05 will be considered significant for all analyses, and 95% confidence intervals (CIs) will be calculated.

The secondary outcome measures will be mean changes in values on four additional metrics: the 100-mm VAS, the K-WOMAC scale, the PGA, and the EQ-5D-5 L. These data will be analyzed using the same methods as for the primary outcome measure. Subgroup analyses will be conducted to determine the effect of sex, age, severity of pain, previous treatment, expected level of treatment, body mass index (BMI), and patterns of cold-heat and deficiency-excess [27] on the efficacy of EM and TIM.

### Adverse events

Adverse events known to follow moxibustion treatment include blisters, redness, itching, burns, and respiratory symptoms. In every visit, adverse events will be reported by participants and examined by the practitioner. The presence of symptoms and their onset, duration, severity, and relationship with adverse events from treatment will be carefully recorded in the CRFs. To avoid potential adverse events, a treatment will be delayed by 3 days if the practitioner considers the participant unsuitable for moxibustion treatment, or if the participant indicates moderate fatigue or abnormal health. Vital signs will also be measured at every visit.

## Discussion

Osteoarthritis is caused by prolonged use of articular cartilage, leading to secondary changes and symptoms [28], and is common in the knee joints [2]. Patients with KOA initially suffer from mild pain, swelling, and cracking. As the disease progresses, patients experience muscular dystrophy, limitation in motion, joint locking, severe pain, and decreased quality of life [29, 30]. Therefore, the objective of treating KOA is to improve the quality of life by providing pain relief and preservation of function.

Moxibustion is a common complementary and alternative intervention that has been used to treat KOA. The thermal stimulation from moxibustion increases microcirculation [31] and promotes metabolism [32] by altering blood flow rate and cell membrane permeability. The heat from moxibustion also stimulates immune cells in the skin tissues, leading to immunomodulatory effects [33]. Moreover, histotoxins, which elicit an anti-histamine response, are secreted at the sites of moxibustion and these have an analgesic effect [34]. Moxibustion also elicits anti-inflammatory effects through enhancing the function of phagocytes [35].

The EM device was developed in an attempt to provide a safer, more controlled, and more convenient alternative to TIM. The EM device consists of heating units and charging equipment, and is adjusted by a timer switch, in which a probe is attached to the skin using medical tape. The heating units have a self-regulating control system that automatically changes the temperature, to mimic the temperature change curve in TIM [36].

Because EM maintains a safe maximum temperature, this reduces the risk of skin burns and ignition of linen and clothing. Furthermore, EM does not produce smoke or fumes, which may cause respiratory discomfort in patients and practitioners, so a separate ventilation system is therefore not required. The EM device is simple to operate and store, allowing more efficient use of human resources. These many advantages of EM motivated us to perform a clinical trial to assess its efficacy and safety in patients with KOA.

To assess the efficacy of EM, we will compare it with TIM, which has been widely used for treating KOA, and with usual care (control). The primary outcome measure will be the mean change in pain on a NRS, because pain is the most common symptom of patients with KOA. The secondary outcome measures will be pain on a 100-mm VAS, the K-WOMAC scale (which distinguishes symptoms of KOA such as pain, stiffness, and physical function), PGA, and the EQ-5D-5 L (which assesses overall improvement and quality of life).

A limitation of this trial is that participants and practitioners will not be blinded. Blinding of practitioners is difficult because the appearance of EM and TIM equipment are very different. Although the eyes of participants will be covered during treatment, it is difficult to completely blind them to smoke and odors. Therefore, an independent researcher who is unrelated to the allocation and treatments will conduct all assessments to decrease possible bias.

Another limitation is that the control group will be given usual care, rather than treatment with a sham device. There is growing use of this approach in pragmatic clinical trials [37, 38]. The internal validity is low, because participants and practitioners are not blinded; however, the external validity is high because the results are more meaningful in clinical settings. In other words, this design more directly addresses the main question of this trial: is EM effective and safe for treating KOA in clinical practice?

The results of our trial will provide important new evidence on the efficacy of EM in reducing the pain and improving the physical function of patients with KOA. It will also test the efficacy of an alternative moxibustion technique, in which there is no smoke and odors, a major problem in the conventional moxibustion that is used by many doctors of traditional Korean medicine [39]. Thus, our trial examines the safety and efficacy of EM as an alternative to conventional moxibustion.

### Trial status

Participant recruitment is still being undertaken. Enrollment started in September 2017 and trial completion is expected to be finished by December 2018.

## Additional file

Additional file 1: SPIRIT 2013 checklist. (DOCX 51 kb)

## Abbreviations

ACR: American College of Rheumatology
ANCOVA: Analysis of covariance
ANOVA: Analysis of variance
BMI: Body mass index
CIs: Confidence intervals
CRFs: Case report forms
EM: Electrical moxibustion
EQ-5D-5 L: European quality of life five dimension five level scale
FAS: Full analysis set
HRQoL: Health-related quality of life
KOA: Knee osteoarthritis
K-WOMAC: Korean version of the Western Ontario and McMaster osteoarthritis index
LOCF: Last observation carried forward
NRS: Numerical rating scale
NSAIDs: Non-steroidal anti-inflammatory drugs
PGA: Patient global assessment
PP: Pre-protocol
SD: Standard deviation
TIM: Traditional indirect moxibustion
VAS: Visual analog scale
WOMAC: Western Ontario and McMaster osteoarthritis index
